# Prevalence of Hepatitis D in the Eastern Mediterranean Region: Systematic Review and Meta Analysis

**DOI:** 10.5812/hepatmon.8210

**Published:** 2013-01-06

**Authors:** Neda Amini, Seyed Moayed Alavian, Ali Kabir, Seyed Hossein Aalaei-Andabili, Seyed Yasser Saiedi Hosseini, Mario Rizzetto

**Affiliations:** 1 Tehran University of Medical Sciences, Students' Scientific Research Centre, Tehran, IR Iran; 2 Baqiyatallah Research Center for Gastroenterology and Liver Diseases, Baqiyatallah University of Medical Sciences, Tehran, IR Iran; 3 Department of Epidemiology, School of Public Health, Shahid Beheshti University of Medical Sciences, Tehran, IR Iran; 4 Center for Educational Research in Medical Sciences, Tehran University of Medical Sciences, Tehran, IR Iran; 5 Division of Gastroenterology, Molinette – University of Turin, Corso Bramante, Turin, Italy

**Keywords:** Epidemiology, EMRO, Hepatitis D, Meta-Analysis, Prevalence

## Abstract

**Background:**

Hepatitis D Virus (HDV) causes the most threatening form of chronic viral hepatitis. To date, there is no overall estimation of HDV prevalence in the Eastern Mediterranean Region Office of WHO (EMRO) countries.

**Objectives:**

To provide a clear estimation of HDV prevalence in the aforementioned region.

**Patients and Methods:**

In the current systematic review, databases such as PubMed, Embase, Web of sciences and Google scholar were searched Until December 2010. The summary estimate of HDV prevalence in the EMRO region was calculated as an average of the pooled infection prevalence of each country weighted by the ratio of the country’s HBV population to the study’s sample size in the survey data analysis.

**Results:**

We included 62 eligible studies. The weighted mean of HDV prevalence in the EMRO region was 14.74% (95% CI: 14.73 – 14.77), 27.8% (95% CI: 27.78 – 27.82), 36.57% (95% CI: 36.55 – 36.59) and 16.44%. (95% CI: 16.42 – 16.46) in asymptomatic HBsAg positive carriers, chronic hepatitis patients, cirrhosis/ hepatocellular carcinoma, and high risk group, respectively. Among the asymptomatic HBsAg positive group, HDV prevalence was increased by years in older patients in Saudi Arabia but its prevalence was decreased in Iran. No specific pattern was seen according to chronological analysis during years among the EMRO countries.

**Conclusions:**

HDV infection is endemic in the EMRO countries and it is more common among patients with severe forms of hepatitis. Due to the high HDV infection rates in the EMRO countries, we recommend blood screening for HDV infection in this region.

## 1. Background

The hepatitis D virus (HDV) was detected by Rizzetto among patients with a severe form of HBV infection in the year of 1977 (1). HDV is a deformed and incomplete delta agent RNA virus which is dependent on HBsAg for transmission and replication (2). HDV leads to fulminant hepatitis and further disease progression among hepatitis B infected patients. The long term co-infection of HBV and HDV is associated with a higher risk of cirrhosis and hepatocellular carcinoma. Around 15% of only HBV infected patients progress to cirrhosis versus up to 80% of HBV and HDV co-infected patients who develop cirrhosis (3). It has been estimated that almost 5% of HBV infected patients have HDV co-infection (4). The epidemiologic distribution of HDV infection differs throughout the world and in countries such as Iran (5) and in the USA varies from region to region (6). Evidences show that HDV is highly endemic in the Middle East area (7).

## 2. Objectives

Since there are few epidemiological studies in the Eastern Mediterranean Region Office of WHO (EMRO) countries and there is not any overall estimation of hepatitis D infection prevalence in this region, we designed a literature review.In order to provide a clear and comprehensive presentation of available data, we decided on a systematic review on epidemiological characteristics and on finding gap knowledge about HDV infection among HBV infected patients of the EMRO countries according to quantitative analysis of available epidemiological data from this region.

## 3. Patients and Methods

### 3.1. Search Strategy

We made an electronic literature search through Scopus, Web of sciences, Google scholar, Embase and two MEDLINE database engines; Pubmed and Ovid using different combinations of following key words “hepatitis D, Delta antigen, HDV, hepatitis delta virus” and the name of the EMRO countries as; Afghanistan, Bahrain, Cyprus, Djibouti, Egypt, Iran, Iraq, Jordan, Kuwait, Lebanon, Libya, Morocco, Oman, Pakistan, Palestine, Qatar, Saudi Arabia, Somalia, Sudan, Syria, Tunisia, United Arab Emirates, and Yemen. The Iranian databases such as MagIran, IranMedex and SID were also searched with relevant English and Persian key words. We searched published/unpublished information until December 2010. Search sensitivity was checked by considering duplicated papers. If the full text of articles were not accessible, e-mails were sent to authors. After one month, if authors did not give a response, informative abstracts were used for data extraction. The articles with no informative abstracts were omitted.

### 3.2. Study Selection

Published studies in English, Persian, and French were eligible if they met the following criteria: 

1) Appropriate study design: cross-sectional, case-control, and case-series or cohort,

2) Studies with clearly stated information about the number of HBsAg positive patients infected with HDV in the EMRO countries. The following represents our exclusion criteria: 

1) Studies with all patients having acute hepatitis B because the pattern of HDV is different in chronic and acute hepatitis.

2) Articles were about the genotypes.

3) All participants were under 15 years old.

4) Papers included HIV positive patients as participants.

The names of the authors or journals had no impact on the decision to exclude or include the articles.

### 3.3. Quality Assessment

A critical appraisal (CA) was done using Epible check list form (8) to evaluate the adequacy of the sample size, design, data collection, and the resultant presentation. Each paper was appraised by two authors individually. Then, the two CA scores of each paper were compared together. If the difference was more than 10 percent, authors negotiated to reach the same CA score. Based on the total CA score, articles were divided into low (< 40%), moderate (40% - 70%), and high (> 70%) quality. Low quality papers were not included in the main analysis except in subgroups analysis according to the papers’ quality.

### 3.4. Data Extraction

Information was entered into Microsoft Office Excel 2007. The name of country, the author’s name, the year of study, the sample size, HBsAg positive frequency, the mean age, and the total prevalence of HDV were extracted. HDV prevalence was calculated in different subgroups consisting of: 1) cirrhotic and hepatocellular carcinoma 2) asymptomatic HBsAg positive carriers consisting of inactive carriers, general population, blood donors and healthy pregnant women 3) chronic hepatitis patients and 4) high risk group including intravenous drug abusers (IDU) and hemodialysis patients. Moreover, standard errors (SE) were calculated using the following formula: SE = √ (P*(1-P)/N) (P = prevalence, N= sample size)

### 3.5. Statistical Analysis

The aggregated prevalence of each country was computed by “metan” command in which is an average of the individual study results weighted by the inverse of their variances using a fix/random model (DerSimonian and Laird) based on the heterogeneity test result that was showed by Q squared, I - squared and Tau - squared statistics. For Q statistics, due to the low power of this test, a minimum cut-off P value of 0.1 was established as a threshold of heterogeneity. I-squared lies between 0% and 100% and Tau-squared showed the variance between studies. The summary estimate of HDV prevalence in the EMRO region was calculated as an average of the pooled infection prevalence of each country weighted by the ratio of the country’s HBV population to the study’s sample size in the survey data analysis. Subgroup analysis was planned depending on the disease pattern (cirrhotic and hepatocellular carcinoma, asymptomatic HBsAg positive carriers, chronic hepatitis patients, and the high risk group) and quality assessment scores (low, moderate, and good). The analysis was performed with Stata 11 software (Stata Corp. LP).

## 4. Results

### 4.1. Search Results

Two hundred and four articles were found in the literature review, 145 of them were potentially related to HDV in the EMRO countries. After reviewing of the abstracts and titles, 38 articles (9-43) were omitted since they were related to the genotypes or were editorial, and review articles. In addition, three studies (42-44) with duplicated data of the same populations were excluded. In addition, sixteen citations were omitted (43-57) as they were not available online, and their abstracts did not provide sufficient information. Although we contacted their authors or publishers, their full texts were not made accessible. Eighteen studies (56-73) with all patients having acute hepatitis B were excluded and three other (74-76) studies were also omitted because all participants were children. Ten (77-86) further studies were omitted because they were categorized in a low quality group. Thus, the 62 (5, 87-143) studies to be included were identified assessing the HDV prevalence in the EMRO countries and fulfilling inclusion criteria. Out of these,19 studies (95-113) were in Iran, nine studies (129-137) in Saudi Arabia, seven studies (5, 119-124) in Pakistan, six studies (89-94) in Egypt, four studies (138-141) in Tunisia and Somalia (125-128) and two studies (142, 143) in Yemen. There was one study of other countries which consisted of: Afghanistan (87), Djibouti (88), Jordan (114), Kuwait (115), Lebanon (116), Morocco (117), and Oman (118). There were no data available from the following countries: Bahrain, Cyprus, Iraq, Libya, Palestine, Qatar, Syria, and United Arab Emirates. Three studies (127-129) had case-control design and the other 59 were cross-sectional.

### 4.2. HDV Infection Prevalence Among Asymptomatic HBsAg Positive Carriers

**Figure 1:**
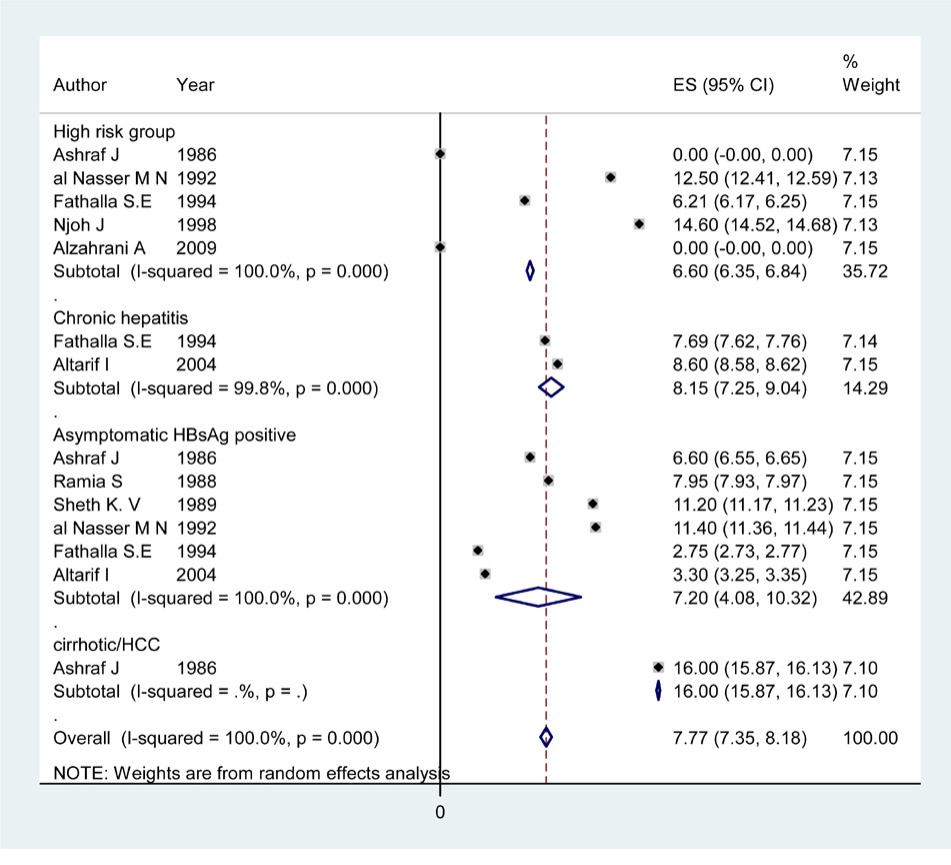
Forest Plot of HDV Infection Prevalence among Different Subgroups in Pakistan

**Figure 2:**
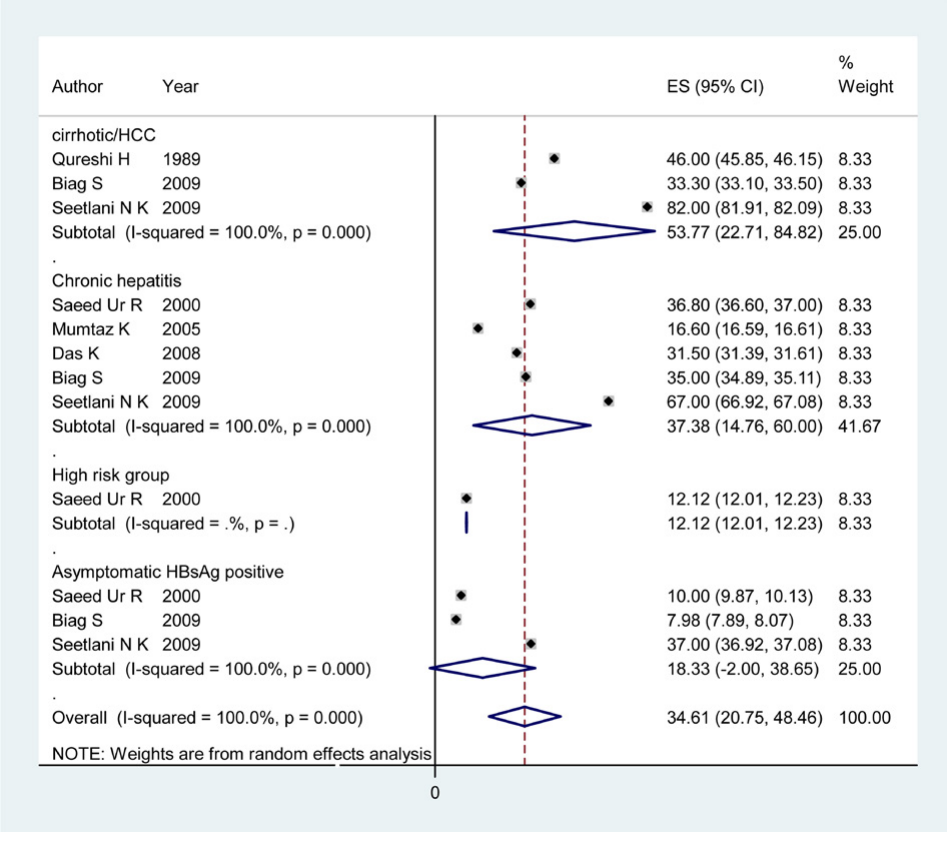
Forest Plot of HDV Infection Prevalence among Different Subgroups in Egypt

**Figure 3:**
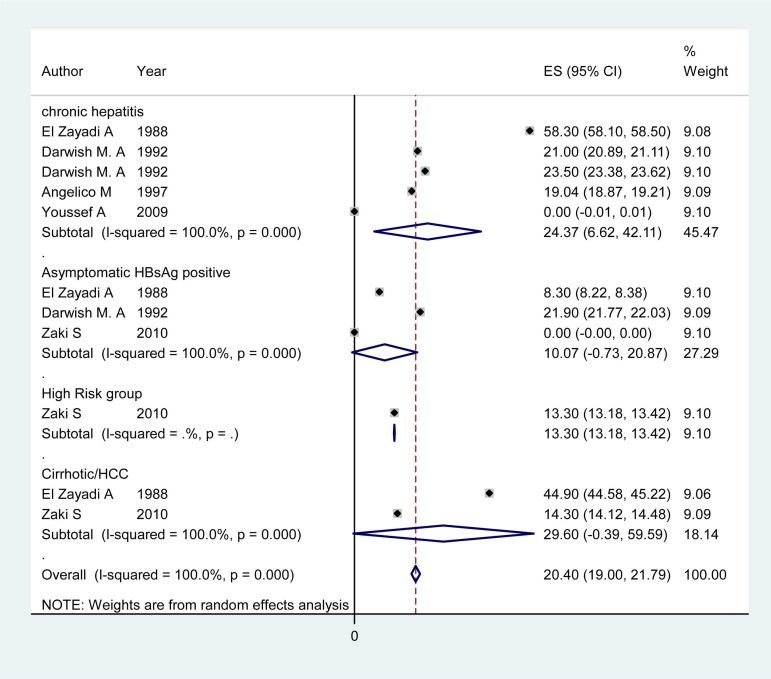
Forest Plot of HDV Infection Prevalence among Different Subgroups in Saudi Arabia

**Figure 4:**
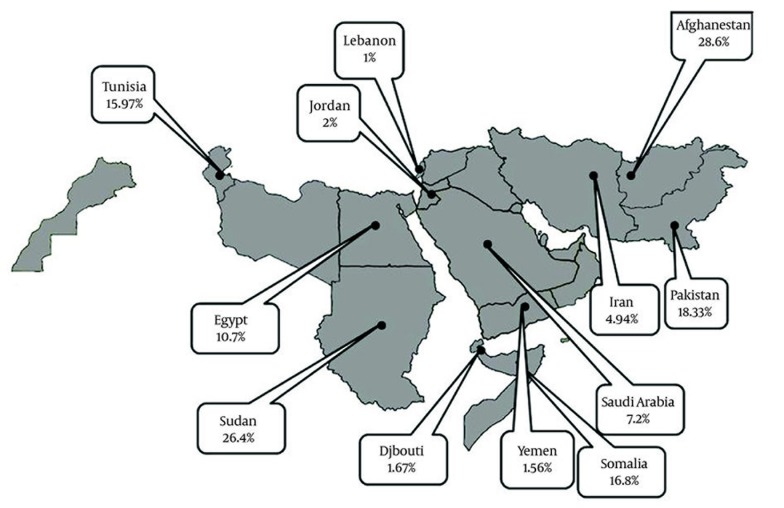
Regional Distribution of Pooled or Individual Prevalence of Hepatitis D Virus Infection among Asymptomatic HBsAg Positive in EMRO Countries

Information about HDV prevalence of asymptomatic HBsAg positive carriers was available from 12 countries (Table 1). The pooled HDV prevalence among this group in the countries with more than one study was 24.6% (95% CI: 23.66 – 29.15) in Sudan, 18.33% (95% CI: -2.00 – 38.65) in Pakistan (Figure 1), 15.97% (95% CI: 9.40 – 22.55) in Tunisia, 10.7% (95% CI: 0.73 – 20.87) in Egypt (Figure 2), 7.2% (95% CI: 4.08 – 10.32) in Saudi Arabia (Figure 3), 4.94% (95% CI: 3.73 - 6.15) in Iran, and 1.56% (95% CI: 1.10 – 2.03) in Yemen. Infection prevalence in the countries for which only one report was available ranged from 1%, 1.67% and 2% in Lebanon, Djibouti, and Jordan to 16.8% and 28.6% in Somalia and Afghanistan. The pooled or individual estimation of HDV prevalence in these countries provided in (Figure 4). According to the survey data analysis method, the HDV prevalence for each country was weighted using the ratio of the country’s HBV population to the study’s sample size. Using this method, the weighted mean HDV prevalence in the EMRO countries was 14.74% (95% CI: 14.73 – 14.77) among asymptomatic HBsAg positive group.

**Table 1:** Characteristics of Studies in Asymptomatic HBsAg Positive Carriers in EMRO Countries

Name of Country	First Author/Publication, y	Quality Assessment Score	Sample Size	HDV Prevalence, %`
**Afghanistan**	Jacobson, Ira M./1985	moderate	7	28.60%
**Djibouti**	E. A. Abbatte/1989	moderate	656	1.67%
** **	El Zayadi, A./1988	moderate	48	8.30%
** **	Darwish, M. A/1992	moderate	41	21.90%
** **	Zaki, S/2010	moderate	6	0
**Iran**	Malekzadeh, R./1989	moderate	158	13.90%
** **	Rezvan, H./1990	moderate	120	2.50%
** **	Amini, S./1993	good	123	2.40%
** **	Habibi, F/2000	moderate	200	9.00%
** **	Karimi, A./2000	moderate	154	1.30%
** **	Hassanjani Roshan, M. R./2004	moderate	546	2.00%
** **	Alavian, S.M./2004	moderate	102	2.00%
** **	Roshandel, G/2007	good	139	5.80%
** **	Amini, S/2007	moderate	79	8.00%
** **	Taghvaei, T/2008	moderate	167	0%
** **	Doosti, A./2009	moderate	200	3.00%
** **	Somi, M. H/2009	moderate	300	4.70%
** **	Hajiani, E/2009	good	1725	3.59%
** **	Ataei, B./2010	good	346	2.80%
** **	Alizadeh, A. H./2010	moderate	48	20.80
** **	Jedary Seifi, S./2010	moderate	355	6.00%
**Jordan**	Toukan, A. U/1987	good	136	2.00%
**Lebanon**	Ramia, S/2007	moderate	107	0.90%
**Pakistan**	Saeed Ur, R/2000	moderate	20	10.00%
** **	Baig, S/2009	moderate	38	7.98%
** **	Seetlani, N.K/2009	moderate	143	37.00%
**Somalia**	Aceti, A/1989	moderate	220	16.80%
**Saudi Arabia**	Ashraf, S. J/1986	moderate	102	6.60%
** **	Ramia, S/1988	moderate	465	7.95%
** **	Sheth, K. V./1989	moderate	490	11.20%
** **	El Nasser, M. N./1992	moderate	256	11.40%
** **	Fathalla, S. E/1994	moderate	182	2.75%
** **	Al Traif, I/2004	moderate	60	3.30%
**Sudan**	Al-Arabi, M. A./1987	moderate	20	25.00%
** **	Hyams, K. C./1989	good	115	27.80%
**Tunisia**	Jenhani, F/1990	moderate	45	33.00%
** **	Triki, H/1997	moderate	650	16.10%
** **	Skouri, H/2004	moderate	139	8.00%
** **	Djebbi, A./2009	moderate	176	6.80%
**Yemen**	El Guneid, A. M./1993	moderate	150	1.33%
** **	Scott, D. A/1990	moderate	112	1.80%

### 4.3. HDV Infection Prevalence Among Patients With Chronic Hepatitis

**Figure 5:**
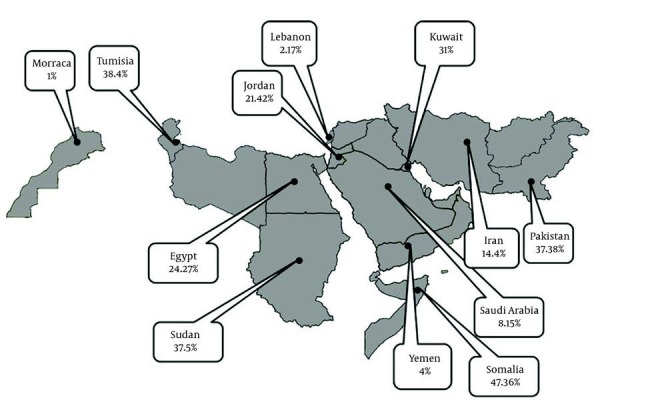
Regional Distribution of Pooled or Individual Prevalence of Hepatitis D Virus Infection among Patients with Chronic Liver Disease at EMRO Countries

Articles of 12 countries revealed information on the HDV infection rate among patients with chronic hepatitis (Table 2). The pooled HDV prevalence was 47.36% (95% CI: 30.24 – 64.49) in Somalia, 37.38% (95% CI: 14.76 – 60) in Pakistan (Figure 1), 24.37% (95% CI: 6.62 – 42.11) in Egypt (Figure 2), 14.4% (95% CI: 7.72 - 21.07) in Iran, and 8.15% (95% CI: 7.25 – 9.04) in Saudi Arabia (Figure 3). The pooled or individual estimation of HDV prevalence among chronic hepatitis patients demonstrated in Figure 5. The weighted mean HDV prevalence was 27.8% (95% CI: 27.78 – 27.82) in the EMRO countries.

**Table 2:** Characteristics of Studies among Patients with Chronic Hepatitis Disease in EMRO Countries

Name of Country	First Author/Publication, y	Quality Assessment Score	Sample Size	HDV Prevalence, %
**Egypt**	El Zayadi, A./1988	moderate	24	58.30%
** **	Darwish, M. A/1992	moderate	51	21.00%
** **	Darwish, M. A/1992	good	51	23.50%
** **	Angelico M/1997	moderate	21	19.04%
** **	Youssef A/2009	moderate	10	0%
**Iran**	Rezvan, H./1990	moderate	5	0%
** **	Alavian, S.M./2004	moderate	155	7.70%
** **	Taghavi, S. A./2008	good	93	9.70%
** **	Somi, M. H/2009	moderate	547	12.70%
** **	Hajiani, E/2009	good	88	45.50%
** **	Alizadeh, A. H./2010	moderate	30	13.30%
** **	Zahedi, MJ/2010	good	196	10.70%
**Jordan**	Toukan, A. U/1987	good	42	21.42%
**Kuwait**	Alkandari, S./1988	moderate	48	31.00%
**Lebanon**	Ramia, S/2007	moderate	92	2.17%
**Morocco**	Rioche, M/1987	moderate	85	1.00%
**Pakistan**	Saeed Ur, Rahman/2000	moderate	22	36.80%
** **	Mumtaz, K./2005	good	8721	16.60%
** **	Das, K./2008	moderate	73	31.50%
** **	Seetlani, N.K/2009	moderate	141	67.00%
** **	Baig, S/2009	moderate	70	35.00%
**Somalia**	Aceti, A/1991	moderate	41	56.10%
** **	Bile, K/1993	moderate	44	38.60%
**Saudi Arabia**	Fathalla, S. E/1994	moderate	52	7.69%
** **	Al Traif, I./2004	moderate	780	8.60%
**Tunisia**	Djebbi, A./2009	moderate	39	38.40%
**Yemen**	el Guneid, A. M./1993	moderate	25	4.00%

### 4.4. HDV Infection Prevalence Among Cirrhotic/HCC Patients and High Risk Group 

Table 3 showed information about HDV prevalence among both cirrhotic/HCC patients and high risk groups. The pooled HDV prevalence among cirrhotic/HCC patients was 53.77% (95% CI: 22.71 - 82.84) in Pakistan (Figure 1), 33.20 (95% CI: 21.64 – 44.76) in Somalia, 30.47 %( 95% CI: 9.76 - 51.19) in Iran and 29.6% (95% CI: -0.39 -59.59) in Egypt (Figure 2). The weighted mean HDV prevalence of patients with cirrhosis or hepatocellular carcinoma was 36.57% (95% CI: 36.55 – 36.59) among nine countries revealing information on the HDV infection rate in cirrhotic/HCC patients. In the high risk group consisting of IDU and hemodialysis patients, the weighted mean HDV prevalence in six countries was 16.44%. (95% CI: 16.42 – 16.46).

**Table 3:** Characteristics of Studies in Cirrhotic/HCC Patients and High Risk Group in EMRO Countries

Name of Country	First Author/Publication, y	Quality Assessment Score	Cirrhotic/HCC Patients		High Risk Group	
			Sample Size	HDV Prevalence, %	Sample Size	HDV Prevalence, %
**Egypt**	el Zayadi, A./1988	moderate	9	44.90%		
** **	Zaki, S/2010	moderate	14	14.30%	30	13.30%
**Iran**	Karimi, A./2000	moderate	71	50.00%	65	2.00%
** **	Alavian, S.M./2004	moderate	23	8.70%		
** **	Makvandi, M/2004	moderate	16	20.00%		
** **	Taghavi, S. A./2008	good			15	26.60%
** **	Hajiani, E/2009	good	88	43.20%		
** **	Jedary Seifi, S./2010	moderate			190	8.94%
** **	Alizadeh, A. H./2010	moderate			7	28.57%
**Jordan**	Toukan, A. U/1987	good	52	51.30%		
**Lebanon**	Ramia, S/2007	moderate	35	0	20	0
**Oman**	Aghanashinikar, P. N./1992	moderate			22	13.63%
**Pakistan**	Qureshi, H./1989	moderate	45	46.00%		
** **	Saeed Ur, Rahman/2000	moderate			33	12.12%
** **	Baig, S/2009	moderate	21	33.00%		
** **	Seetlani, N.K/2009	moderate	78	82.00%		
**Somalia**	Bile, K/1991	moderate	23	39.10%		
** **	Aceti, A/1991	moderate	11	27.30%		
**Saudi Arabia**	Ashraf, S. J/1986	moderate			6	0
** **	Ashraf, S. J/1986	moderate	30	16.00%		
** **	al Nasser, M. N./1992	moderate			50	12.50%
** **	Fathalla S. E/1994	moderate			177	6.21%
** **	Njoh, J./1998	moderate			75	14.60%
** **	Alzahrani, A. J./2009	moderate			41	0
**Tunisia**	Jenhani, F/1990	moderate	114	21.00%		

### 4.5. HDV Prevalence According to Quality Assessment Score

According to the quality assessment score, ten articles were categorized in the good quality group (5, 29, 91, 97, 99, 102, 109, 111, 113, 114). In this group, HDV prevalence was 16.25%, 16.60%, 44.32% among asymptomatic HBsAg positive carriers, chronic hepatitis patients and cirrhotic/HCC patients, respectively. HDV prevalence in moderate quality group consisted of 52 articles (87-90, 92-96, 98, 100, 101, 103-108, 110, 112, 115-145) among asymptomatic HBsAg positive carrier was 14.02% and among chronic hepatitis patients was 28.73%. In cirrhotic/HCC and high risk group, HDV prevalence was 36% and 16.44%. In low quality group, we had ten articles (77-86). The HDV prevalence was 44.50%, 22.67%, and 59.44% among asymptomatic HBsAg positive carriers, chronic hepatitis, and cirrhotic/HCC patients, respectively. We had no data among the high risk group in low quality and good quality articles.

### 4.6. Chronologic Changes of HDV Prevalence 

To detect the HDV prevalence pattern over the years in the EMRO countries, we sorted HDV infection prevalence by year for each country. This analysis also was repeated for the whole EMRO region. The data was not shown; however, no specific pattern was seen. The overall estimation for three age categories (< 20, 20-30, > 30) was calculated in different populations (asymptomatic HBsAg positive persons, chronic hepatitis, cirrhotic and hepatocellular carcinoma). Due to lack of information for most countries, this table was not shown in the results. The only recognizable patterns was the increase of HDV prevalence in Saudi Arabia by year among the asymptomatic HBsAg positive group and decrease of infection prevalence by year in Iran of the same group.

## 5. Discussion

The result of this study revealed HDV prevalence was 15% and 26% among asymptomatic HBsAg positive carriers and chronic hepatitis patients, respectively. This result indicates HDV infection is endemic in the EMRO region. In addition, the most prevalence of HDV infection was in African countries of EMRO regions such as Egypt, Sudan, Tunisia and Somalia. As a result, North Africa must be considered as a high HDV prevalence area in addition to central Africa, southern America, and Mediterranean countries since they were mentioned in the previous studies (146). The comparison of HDV prevalence between different WHO regions was demonstrated in Table 4. In the African region, the infection prevalence is near to mean of the HDV prevalence in the EMRO region. In other regions, some countries such as Brazil (147), Venezuela (148), Turkey (149), and Mongolia (150) have higher HDV infection prevalence in comparison with the EMRO countries. Our analysis showed that a cirrhotic/HCC group had the most HDV infection prevalence when correlated with a previous study which has consistently shown most patients with HBV and HDV co-infection have a more severe progression to cirrhosis and HCC (151, 152). Furthermore, HDV infection prevalence was calculated to be 16% among IDU and hemodialysis group (high risk group) that is less common in comparison with asymptomatic and chronic hepatitis patients. This result is in contrast with previous studies (146, 153) and indicates that IDU and people exposed to blood and its products were at a high risk of acquiring HDV infection. This result may be due to our limited existing data among IDU and hemodialysis group. The analysis according to the quality assessment score showed that HDV prevalence was overestimated among asymptomatic HBsAg positive carriers in low quality articles. Also, this prevalence was underestimated among chronic hepatitis patients. Furthermore, the subject of HDV infection prevalence in good quality articles was closed to overall estimation. We had some limitations in our review such as lack of good coverage caused by hand searching the library and dissertations. Also, for most of the countries, our literature review was limited to English. As a result, the information in native languages was not operational. Another limitation was related to HDV detection method which was ELISA for most articles. Hence, the confirmation of ongoing HDV infection by PCR testing of HDV RNA was missing. The impact of this lack of information is that patients with and without active delta infection cannot be differentiated. One of our goals in this systematic review was the calculation of a pooled estimation for possible risk factors to identify the most important routes of HDV transmission in the EMRO region. However, the numbers of articles that revealed information in this field were rare. Therefore, we could not perform that analysis. Many articles were done before the year 2000 and some countries had one or two HDV prevalence estimations in close time points. Due to lack of information, a recognizable pattern of HDV prevalence was not detectable during recent years. In addition, most studies were performed on young adults (30-50) therefore; we could not access the impact of age on HDV acquisition in the EMRO region. One of the strengths of this study was doing the survey data analysis in addition to usual ‘Meta’ command that helps increase generalizability of results to the whole population. Twenty two countries (total population is 798,782,000) are in the EMRO region and the data from 92% (738,901,000) of this population was used in our analysis. In conclusion, the HDV infection is endemic in the EMRO countries; and this prevalence is very high in African part of the EMRO region. Furthermore, it is more common among patients who have a severe form of hepatitis. Due to these results, we would recommend blood screening for HDV infection. Furthermore, new surveys are needed to detect infection prevalence in different points of time and to provide the updated data about HDV prevalence in the EMRO region countries.

**Table 4:** Correlation Among HBeAg, ALT, and HBV-DNA

Author (Citation)	Country	Prevalence, %	Sample Size, Target Population	Neighbors City in EMRO Region, Prevalence
**African Region**				
Nwokediuko S (154)	Nigeria	15.60%	Asymptomatic HBsAg positive	Libya (no data)
Rapicetta M (155)	Ethiopia	5.80%	Asymptomatic HBsAg positive	Sudan (26.40%)
Foupouapouognigni Y (156)	Cameroon	17.60%	Asymptomatic HBsAg positive	None
**Americas Region**				
Fonseca JC (147)	Brazil	34.40%	Asymptomatic HBsAg positive	None
Hadler S. C (148)	Venezuela	34.00%	Asymptomatic HBsAg positive	None
**South-East Asia Region**				
Kim H. S (157)	Korea	0.32%	Chronic hepatitis	None
Chakraborty P (158)	India	8.10%	Chronic hepatitis	Pakistan (37.38%)
**European Region**				
Degertekin H (149)	Turkey/middle east	27.10%	Meta analysis	Iran (4.94%)
Gaeta GB (6)	Italy/Europe	8.30%	Chronic hepatitis	None
**Western Pacific Region**				
Chen X (159)	China	3.16%	Asymptomatic HBsAg positive	Afghanistan (28.60%)
Tsatsralt-Od B (150)	Mongolia	43.00%	Chronic hepatitis	None
